# Epidermoid cyst of the tonsil: a rare finding

**DOI:** 10.11604/pamj.2019.34.4.18193

**Published:** 2019-09-02

**Authors:** Rachida Bouatay, Sondos Jellali, Nouha Abdejelil, Jamel Koubaa

**Affiliations:** 1 Department of Otorhinolaryngology and Head and Neck Surgery at “Fattouma Bourguiba” Hospital of Monastir, Tunisia; 2 Pathology Department at “Fattouma Bourguiba” Hospital of Monastir, Tunisia

**Keywords:** Epidermoid cyst, tonsil, surgery

## Abstract

Epidermoid cysts (ECs) are benign lesions that can be encountered throughout the body, but with a low incidence in the head and neck (1.6 to 7%). In oral cavity, the most common affected site is floor of the mouth, but tonsillar location remains extremely rare (less than 0.01%). Here we present an epidermoid cyst of the right palatine tonsil which was incidentally detected in a patient who consulted for a chronic headache.

## Introduction

Epidermoid cysts (ECs), also named epidermal, epithelial, keratinous, sebaceous, or infundibular cysts are benign lesions developing from abnormal epithelial components of ectodermal tissue formed during the fetal period (congenital cysts), or implanted epithelium arising after surgery or trauma (acquired cysts) [1]. They are benign lesions that can be encountered throughout the body, but with a low incidence in the head and neck (1,6 to 7%) [2]. In oral cavity, the most common affected site is floor of the mouth, but tonsillar location remains extremely rare (less than 0.01%). Treatment of epidermoid cysts of the head and neck is surgical and can be intraoral or extra oral according to the localization and the size of the lesion [3]. Here we report a case of epidermoid cyst arising in the tonsil which was encountered as an incidental finding in the patient.

## Patient and observation

**Figure 1:**
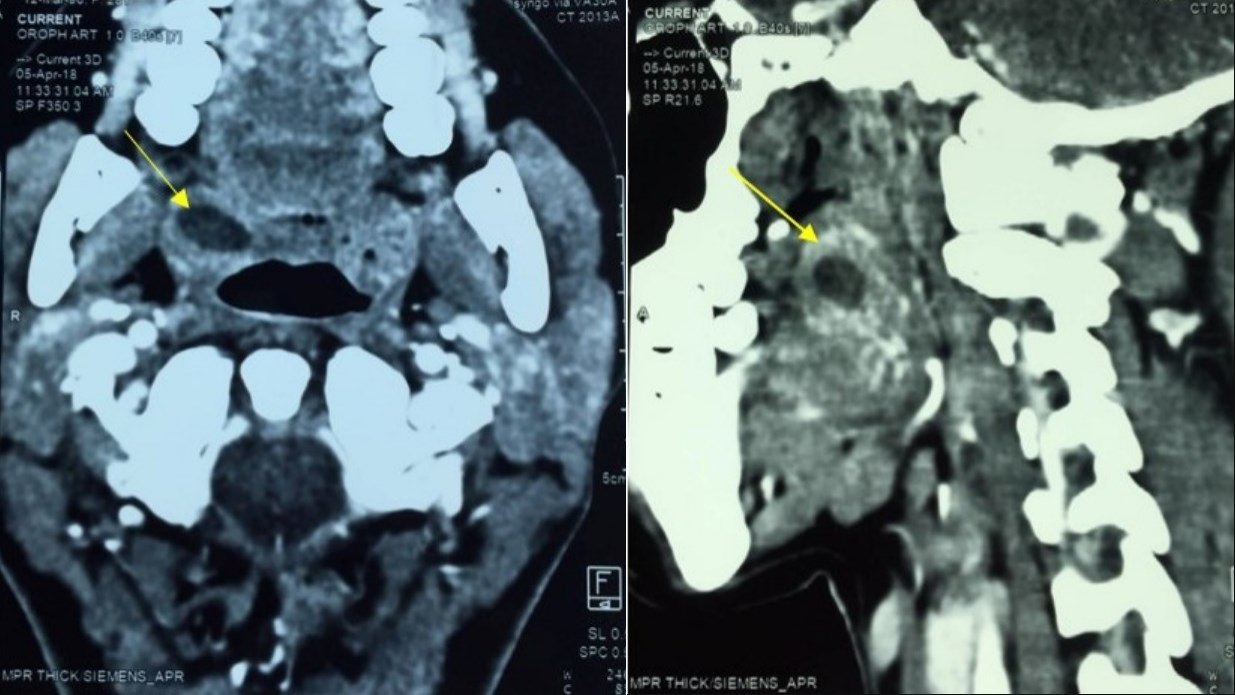
Axial injected computed tomography section of the oropharynx showed an hypo dense mass of the right palatine tonsil with peripheral enhancement

**Figure 2:**
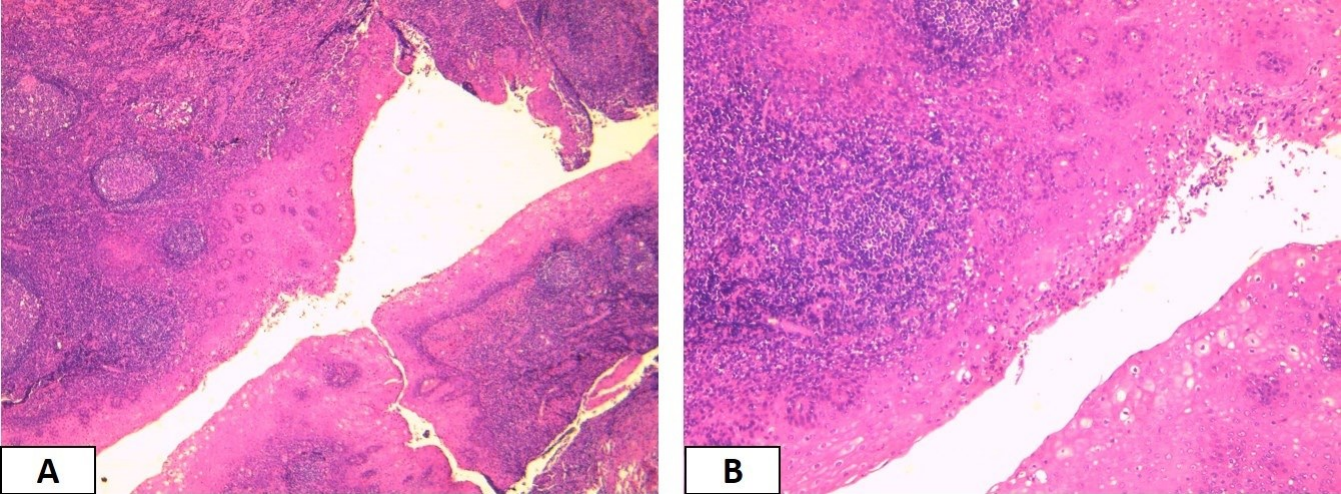
Cystic cavity within the tonsillar tissue lined with keratinized epithelium HE X 40 (A) and HE X 100 (B)

A 28-year-old female with no pathological history, consulted for chronic headache without a history of recurrent tonsillitis. On physical examination the palatine tonsils were hypertrophied and symetric, the throat isthmus was symmetrical. There were no palpable neck masses or other findings on head and neck examination. An injected cerebral computed tomography performed, showed cystic formation of the right palatine tonsil which enhanced in peripheral with a center of fluid density (Figure 1). The patient underwent tonsillectomy under general anesthesia for diagnostic purpose. Histopathologic examination showed a cystic formation lined by a squamous epithelium surmounted by parakeratotic hyperkeratosis. It contains neutrophils in exoxtosis and rests on lymphoid tissue (Figure 2). These findings confirmed the diagnosis of epidermoid cyst of the tonsil. The patient was discharged without any postoperative complications; her follow-up after 24 months was entirely uneventful.

## Discussion

Epidermoid cysts are defined as benign lesions that are histologically characterized by cystic spaces lined by squamous epithelium [1]. Among the different types of cyst which can arise in tonsils, the tonsillar retention cyst is the most common type while epidermoid cyst, lymphoepithelial cyst and hydatid cyst are rare causes of tonsillar cyst [4]. The cysts in oral cavity are termed as “epidermoid” if they are enclosed and lined by stratified squamous epithelium only. If the wall of the cyst contains skin adnexal structures, they are termed as “dermoid cyst” and if they include the tissues from ecto, endo or mesoderm like muscle, bone, cartilage or fat, they are called “teratoid cyst” [5]. They can be of two types: congenital or acquired, which are similar both clinically as well as histologically [6]. The etiology of epidermoid cysts are varied and noted from hormonal influence during puberty to abnormal inclusion of cells during surgery/trauma or development from the epithelia remnants isolated during the closure of first and second branchial arches in the midline [7].

These cysts can be associated with certain hereditary syndromes like Gardner syndrome caused by mutations in the adenomatous polyposis coli gene, or in Lowe syndrome, an X-chromosomal oculo-cerebral-renal disorder caused by mutations of the OCLR1-gene (Oculo Cerebro Renal Lowe syndrome) [1]. They can occur in any age group, starting from birth (the congenital type) to 72 years. Most patients are in the range between 15 to 35 years, with male preponderance. Our patient was a 28- year-old female [4]. In oral cavity, the sites from which ECs can arise are floor of mouth or the labial, lingual or buccal mucosa [8]. Oral cavity accounts for about 1,6% [9]. They usually present as asymptomatic painless slow growing mass [4,5]. That was the case for our patient since EC presented as an incidental finding during headache assessment. The differential diagnosis to be considered for tonsillar hypertrophy include tumors of parapharyngeal space, infectious etiology, dermoid cyst, lymphoepithelial cyst, papilloma and tonsillar carcinoma. Histopathologically we can easily differentiate these entities, hence gross and microscopical examination of every resected tonsillar mass is important. Histopathology is the gold standard to rule out malignancy and to confirm the benign nature of tonsillar epidermoid cyst [1,7,10].

Diagnosis is based on imaging and can be aided by fine needle aspiration in case of symptomatic tonsillar mass, followed by excision biopsy. Imaging studies can provide some diag¬nostic information about epidermoid cyst. On CT scan, epidermoid cyst should appear as a well-circumscribed, low-density, unilocular mass. Typically, it is predominantly fluid-attenuated. On magnetic resonance imaging, the appearance of EC is variable according to their fluid contents and protein density. Often, they exhibit a low signal intensity with T1 sequences and a high signal intensity with T2 sequences [11,12]. Treatment for these lesions is surgical excision of the cyst or tonsillectomy [13]. It should be excised without opening because its contents could have an irritating effect on the surrounding fibrovascular tissue. A tonsillectomy was performed in our patient. The cyst was excised within its capsule and the follow-up during 24 months was entirely uneventful. Recurrence after surgery is rare [10]. Recurrence rate of ECs is reported to be very less with good prognosis. Malignant transformation has been considered to be extremely rare for head and neck and was reported to have an incidence of 0.5% [5,9].

## Conclusion

The importance of this rare case report is to highlight the rarity of intratonsillar EC presented as an incidental finding in our case and the need for histopathological examination to differentiate it from the tonsillar neoplasia due to similar appearance.

## Competing interests

The authors declare no competing interests.
